# An Exploration of a Synthetic Construction Land Use Quality Evaluation Based on Economic-Social-Ecological Coupling Perspective: A Case Study in Major Chinese Cities

**DOI:** 10.3390/ijerph17103663

**Published:** 2020-05-22

**Authors:** Xufeng Cui, Sheng Yang, Guanghong Zhang, Bin Liang, Fei Li

**Affiliations:** 1 School of Business Administration, Zhongnan University of Economics and Law, Wuhan 430073, China; cxf@zuel.edu.cn (X.C.); 201811080234@stu.zuel.edu.cn (S.Y.); 2 Development and Rural Innovation, Wageningen University and Research, Wageningen 6709PH, The Netherlands; bin.liang@wur.nl; 3 School of Information and Safety Engineering, Zhongnan University of Economics and Law, Wuhan 430073, China

**Keywords:** CLUQ, ECO model, evaluation, cities, obstacle

## Abstract

Recently, with the rapid increase of urban population and industrial agglomeration, the price of construction land has increased, and construction land has become increasingly scarce. Therefore, how to improve the construction land use quality (CLUQ) becomes more and more important. The purpose of the study is to evaluate CLUQ in China’s major cities and to analyze the dominant obstacle factors for quality improvement in order to provide policy advice for construction land management. This study adapts the data from 2014 to 2016 and constructs the evaluation framework of CLUQ involving economic quality, social quality, and ecological quality of construction land to evaluate and analyze CLUQ with the synthetic evaluation model, coupling evaluation model, and obstacle diagnosis model (ECO model). This study shows that the synthetic CLUQ of 23 cities out of 36 major cities in China shows a general increasing state. The economic quality of 26 cities out of 36 major cities in China has increased, while the social and ecological quality of 20 out of 36 major cities in China has decreased. In terms of spatial characteristics, the synthetic quality in the east and southwest of China is relatively high; in terms of spatial trend, the synthetic quality in longitude increases from west to east, and it shows an inverted U-shaped state in latitude. Moreover, economic development is the main obstacle factor for the improvement of CLUQ in Hohhot, Lanzhou, Urumqi, and Changchun. Social development results in the CLUQ lagging in Beijing, Guiyang, Shanghai, Xining, and Chongqing. Ecological development has a negative impact in that of Harbin, Qingdao, and Wuhan. Furthermore. The improvement of CLUQ lies in the coupling and coordinated development of economic, social, and ecological quality. For those with a low coupling degree, the targeted suggestions are given for different types based on city’s quadrant distribution.

## 1. Introduction

Construction land plays an important space and function in urban production and life [1]. Because of the growth of urban population and industrial agglomeration, the price of construction land increases rapidly, and urban construction land is scarcer [2]. At present, in the process of industrialization and urbanization, there are two significant problems in the use of construction land. On the one hand, land use congestion has caused many serious environmental and traffic problems [3,4]; on the other hand, there is some construction land idle [5,6]. These problems are closely related to construction land use. It is important to figure out how to evaluate construction land use quality (CLUQ) scientifically and how to effectively improve construction land use quality.

There have been many studies on agricultural land use quality evaluation, but the research on CLUQ is insufficient; evaluation framework of land use efficiency can provide reference for CLUQ. There are two kinds of framework for evaluating land use efficiency: the single index and the synthetic framework. Single index methodology defines land use efficiency as the economic output from per unit area [7]. Synthetic framework methodology mainly includes economic-social-ecological framework [8,9] and economy-society-ecology-equity framework [10]. Liu et al. [11] established a new framework of land use efficiency from the coordination among grain production, economic development, and ecological protection. Yuan et al. [12] set up the index system of land use efficiency from three aspects of economic, social, and ecological benefits, and calculated the land use efficiency of mining cities in Western China with the improved entropy method. In terms of evaluation methods, a large number of scholars study urban land use by studying urban land use efficiency, so as to put forward effective land use policies to achieve sustainable development of the city [13]. Data envelopment analysis (DEA) [14,15,16], stochastic frontier analysis (SFA) [17], the slack-based measure (SBM) [18,19], AHP [20], the entropy weight method [21], TOPSIS method [22], grey correlation analysis [23], the principal component analysis [24], scale-adjusted metropolitan indicators (SAMIs) [25], bipolar measurement [26], etc., are all applied in the process of evaluation and modeling by international researchers. Among them, Jiao et al. employed the scale-adjusted metropolitan indicators (SAMIs) to assess urban land use efficiency [25]; Zhang et al. used bipolar measurement to evaluate urban land use efficiency with interacting criteria [26]; Kuang et al. used a slack-based measure (SBM) model with undesirable outputs, boxplot, kernel density estimation, and Tobit regression model to evaluate the efficiency of cultivated land use in China [27], and Yang et al. used theoretical analysis, data envelopment analysis, principal component analysis, the coordination coefficient method, and four-quadrant analysis to study urban land use efficiency [28]. In terms of research scale, there are scales on provinces [29], cities [30], urban agglomerations [31,32], Chinese prefecture-level cities [33], high-tech zones in China [34], etc. There are also many studies to analyze land use efficiency for specific land use types, such as cultivated land [35,36], construction land [37], industrial land [38,39], and tourism land [40]. The existing research pays less attention to the evaluation of CLUQ and mainly uses a single evaluation method, so it is of importance to focus on the construction of the evaluation framework of CLUQ and the application of synthetic model.

Based on the above analysis, this study takes 36 major cities in China as the research objects and uses the synthetic evaluation model, coupling evaluation model, and obstacle diagnosis model (ECO model) for analysis. Firstly, the economic, social, and ecological data of the above regions in 2014, 2015, and 2016 are selected to build a synthetic evaluation model of CLUQ for quality evaluation. Then the coupling evaluation model is used to calculate the coupling degree (CD) of the system of CLUQ, and the cities are classified into four groups by the quadrant chart method. After that, the obstacle diagnosis model is used to determine dominant obstacle factors of CLUQ. Finally, combined with quadrant chart analysis, we put forward differentiation construction land use policies, which can provide decision-making reference for improving the CLUQ in the Chinese major cities.

## 2. Data

### 2.1. Study Area

The research object of this paper is 36 main cities in China, namely, the provincial capital cities and above, excluding Hong Kong, Macao, and cities in Taiwan. These cities are the important nodes in China’s urban systems. They have a strong radiation effect on the surrounding cities, so that is why this study selects them as research objects.

### 2.2. Data Source

The data in this paper are mainly collected from “*China City Statistical Yearbook*”, “*China Environmental Statistical Yearbook*”, and statistical yearbooks of provinces and cities. Due to the lack of relevant data in Hong Kong, Macao, and Taiwan, the above regions are not included. The data about permanent population at the end of the year is from the statistical yearbook of each city. The data of PM2.5 annual average concentration is from the “*China Environmental Statistical Yearbook (2015–2017)*” [41,42,43]. The data about the GDP of secondary and tertiary industries, built-up area, fixed assets investment, general public budget revenue, land area, number of health institutions, urban road area and the green coverage area of built-up area were collected from the “*China City Statistical Yearbook (2015–2017)*” [44,45,46].

A descriptive statistical analysis of the collected data is shown in Table 1. Table 1 indicates that there are obvious differences between the cities in China, such as in the GDPs per area (the maximum value is 280,968,500,000 Chinese yuan (RMB)/km^2^, the minimum value is 36,671,590,000 RMB/km^2^, and the standard deviation is 52,331,560,000 RMB/km^2^) and the green coverage ratios of built-up area (the maximum value is 61.58%, the minimum value is 25.5%, and the standard deviation is 5.49%). The development of cities demonstrates a state of unevenness, so it is necessary to evaluate differences in construction land use efficiency, which is valuable for sustainable city land use.

## 3. Methodology

**Figure 1:**
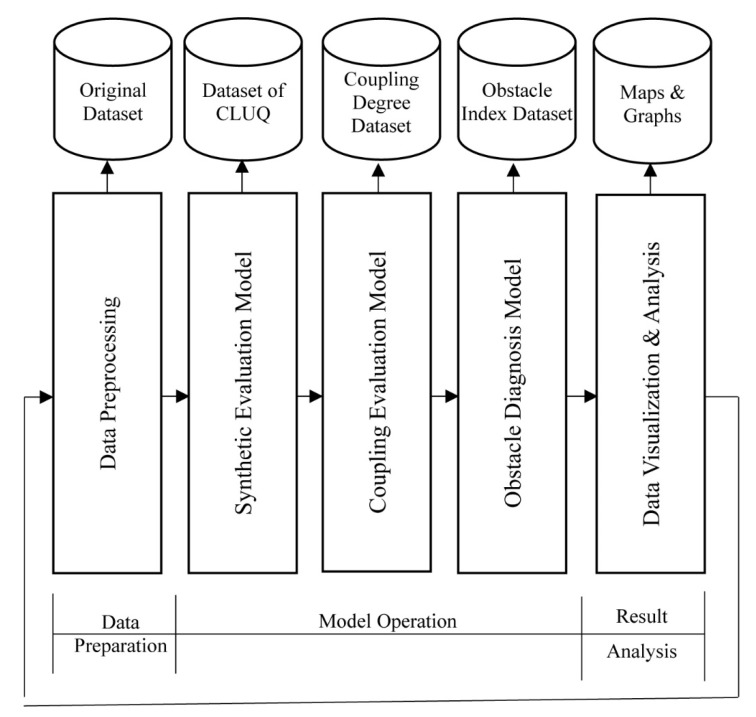
The overall procedure of synthetic evaluation model, coupling evaluation model, and obstacle diagnosis model (ECO model).

ECO model is constructed to execute the analysis of CLUQ (Figure 1). It is divided into 3 stages and includes 5 Modules. The 5 Modules include the data preprocessing, synthetic evaluation model, coupling evaluation model, obstacle diagnosis model, data visualization, and analysis. Among them, the synthetic evaluation model is used to evaluate CLUQ. On the foundation of synthetic evaluation result dataset, the coupling evaluation model analyzes the coupling degree between subsystems so as to judge the coupling level of the system. Obstacle diagnosis model is used determine the dominant obstacle degree based on the coupling degree and quadrant analysis.

### 3.1. Data Preprocessing

*Z*-score normalization method is used for data preprocessing. *Z*-score normalization, also known as standard deviation standardization, gives the meaning and standard deviation of the original data to standardize the data. The processed data conforms to the standard normal distribution, that is, the mean is 0, the standard deviation is 1, and its conversion function is

(1) $$\gamma_{\mathrm{ij}}{}^{*}=\frac{\gamma_{\mathrm{ij}}-\mu (\gamma_{j})}{\sigma (\gamma_{j})}$$

where γ*_ij_ represents standardized value of *γ_ij_, γ_ij_* represents the raw value of the *j*th indicator in *i*th city, *μ(γ_j_)* indicates the mean of *γ_j_,* and σ(*γ_j_*) indicates the standard deviation of *γ_j_*.

### 3.2. Synthetic Evaluation Model

Construction land is an essential spatial support for urban development. The sustainable development of the urban economy, society, and ecology is closely related to the use of construction land. At the same time, people pursue the maximum benefit from construction land with the least land investment. Therefore, the evaluation system of CLUQ should cover the economic, social, and ecological aspects of development. The land user can benefit more from the land, such as to obtain better economic benefits, to create a good ecological living environment, and to build a harmonious social environment, by implementing reasonable land-use methods. From this point of view, after dealing with the scientific rationality and availability of index selection, this paper will construct the evaluation framework of CLUQ from the three criteria levels of economic quality, social quality, and ecological quality of construction land use by combining the previous studies in Table 2.

The economic quality of construction land use mainly refers to the value that can be used by human beings, which is the input-output of land. Land input is measured by fixed-asset investment; the land output is measured by GDP, and fiscal revenue is measured by general public budget revenue.

The social quality of construction land use mainly refers to the satisfaction of social needs in the process of construction land use, including living space, traffic conditions, and health services. Therefore, the per capita urban road area is selected to measure the satisfaction degree of people’s demand for transportation; the per capita built-up area is selected to measure the satisfaction degree of people’s living space and demand, and the coverage rate of social health institutions is selected to measure the satisfaction degree of health service demand.

The ecological quality of construction land use refers to the impact and improvement degree on the ecological environment in the process of construction land use. The research plans to measure the aspects of ecological space, ecological structure, and environmental health select the per capita ecological land area, the green coverage rate of built-up area, and PM_2.5_ annual average concentration index. The per capita ecological land area and green coverage rate of the built-up area are the direct indicators reflecting the level of urban green space construction and are the effective indicators representing the ecological structure. PM_2.5_ annual average concentration is an effective index to measure urban environmental health.

In the evaluation framework of CLUQ, the formulas of each index are calculated as follows: the GDP per area means GDP of secondary and tertiary industries over built-up area; the fixed asset investment per area means fixed assets investment over built-up area; the financial income per area represents general public budget revenue over land area; the coverage ratio of community service institutions means number of health institutions over land area; the per capita urban road area means urban road area over permanent population at the end of the year; the per capita built-up area means built-up area over permanent population at the end of the year; the per capita ecological land area means the green coverage area of built-up area over permanent resident population at the end of the year; the green coverage ratio of built-up area means the green coverage area of built-up area over built-up area; and the annual average concentration of PM_2.5_ means the average PM_2.5_ concentration of the city in one year. Some other details are shown in Table 2.

In the evaluation framework, there are differences in the dimension and order of magnitude of each index. If the original data is used for calculation and analysis directly, the role of the index with higher order of magnitude will be highlighted, and the role of the index with a lower order of magnitude will be weakened. Therefore, to make the evaluation results stable and reliable, it is necessary to standardize the original data, scale the data according to the scale, and map it to a certain range. In this study, we use *Z*-score standardization method to standardize the data. Then value of CLUQ can be computed as follows:

(2) $$\eta_{i}=\sum_{j=1}^{n}\gamma {*}_{ij}\cdot W_{j}$$

where *η_i_* indicates the value of CLUQ in *i*th city, *W_j_* indicates the weight of *j*th indicator. Because of the same importance of all indicators, the same weight is given to each indicator.

In the process of research, the equal weight method and entropy weight method were included in the model calculation as the index weight calculation method, respectively. The results of entropy weight method show that the fitting degree of realistic land use was relatively low; because the entropy weight method uses “degree of dispersion” of the data to calculate the importance of data, the greater the degree of dispersion of the index, the greater the weight of the index. The importance of indicators in reality is not taken into account, while construction land use quality is closely related to the realistic land use. The output of the model driven by equal weight method fits well with reality. At the same time, we also verified this result by consulting many experts, so we choose the equal weight method as the calculation method of index weights. For a more comprehensive presentation of the calculation process, we reported the process and result of entropy weight method in Table S1 from the Supplementary Materials.

### 3.3. Coupling Evaluation Model

The coupling evaluation model is constructed to evaluate the coordination of economic, social, and ecological subsystems. Considering negative results of *Z*-score standardization, a novelty formula is constructed as follows:

(3) $$\Gamma_{i}(\xi_{1}(\eta_{i}),\xi_{2}(\eta_{i}),\xi_{3}(\eta_{i}))=\frac{{\left(\xi_{1}(\eta_{i})-\xi_{2}(\eta_{i})\right)}^{2}+{\left(\xi_{2}(\eta_{i})-\xi_{3}(\eta_{i})\right)}^{2}+{\left(\xi_{1}(\eta_{i})-\xi_{3}(\eta_{i})\right)}^{2}}{\sum_{k=1}^{3}\xi_{k}(\eta_{i})\cdot 3}$$

where Γ*_i_* indicates the coupling degree (abbreviately named as CD) of subsystems. *ζ_i_* indicates the value of CLUQ of each subsystem, such as economic, social, and ecological subsystems.

### 3.4. Obstacle Diagnosis Model

Based on Synthetic evaluation model, obstacle diagnosis model is used for analysis of the obstacle factors of CLUQ.

(4) $$\Theta_{\mathrm{ij}}=\frac{(1-\gamma^{*}{}_{ij})\cdot W_{j}}{\sum_{j=1}^{n}(1-\gamma^{*}{}_{ij})\cdot W_{j}}$$

(5) $$\Psi_{ik}=\sum_{j=1}^{n}\Theta_{ij}$$

where Θ indicates obstacle index of γ*_ij_, and Ψ*_ik_* indicates obstacle degree of *k*th subsystem in *i*th city.

### 3.5. Data Visualization and Analysis

GIS spatial analysis and quadrant analysis methods are employed for data visualization and analysis. In order to map the spatial characteristics of CLUQ, inverse distance weight method (IDW) is used to interpolate the data based on the ArcGIS 10.2 software platform (Environmental Systems Research Institute Inc., Redlands, CA, USA). IDW is a data interpolation method with a linear combination of sample data [59]. “Inverse″ means predicting unknown point value by the weight, which is inversely proportional to distance [60].

**Figure 2:**
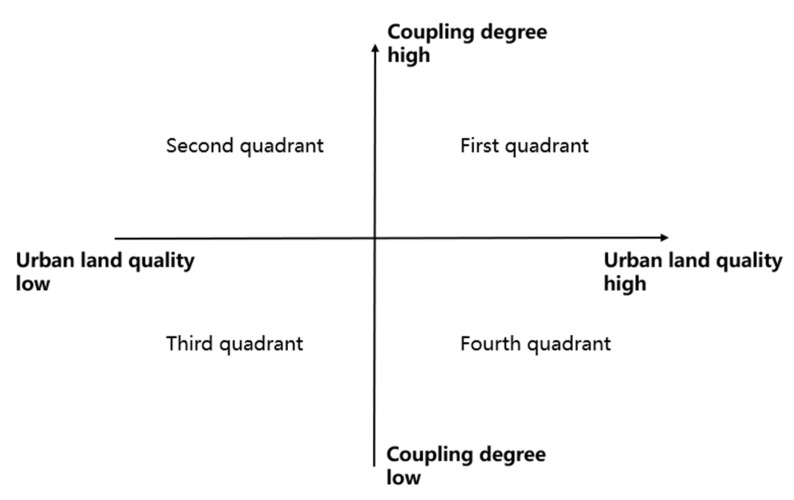
Evaluation value and coupling coordinate axis of urban construction land use quality (CLUQ).

To visualize the relationship between the coupling degree and CLUQ, the cartesian coordinate system is used (Figure 2). The abscissa represents the synthetic evaluation value of CLUQ, while the ordinate represents coupling degree among the three subsystems, it can intuitively show the coupling degree of each city and the relative situation of CLUQ. It can show in which quadrant the city is in CLUQ-CD coordinate system.

## 4. Results and Discussion

### 4.1. Synthetic Evaluation of CLUQ

**Figure 3:**
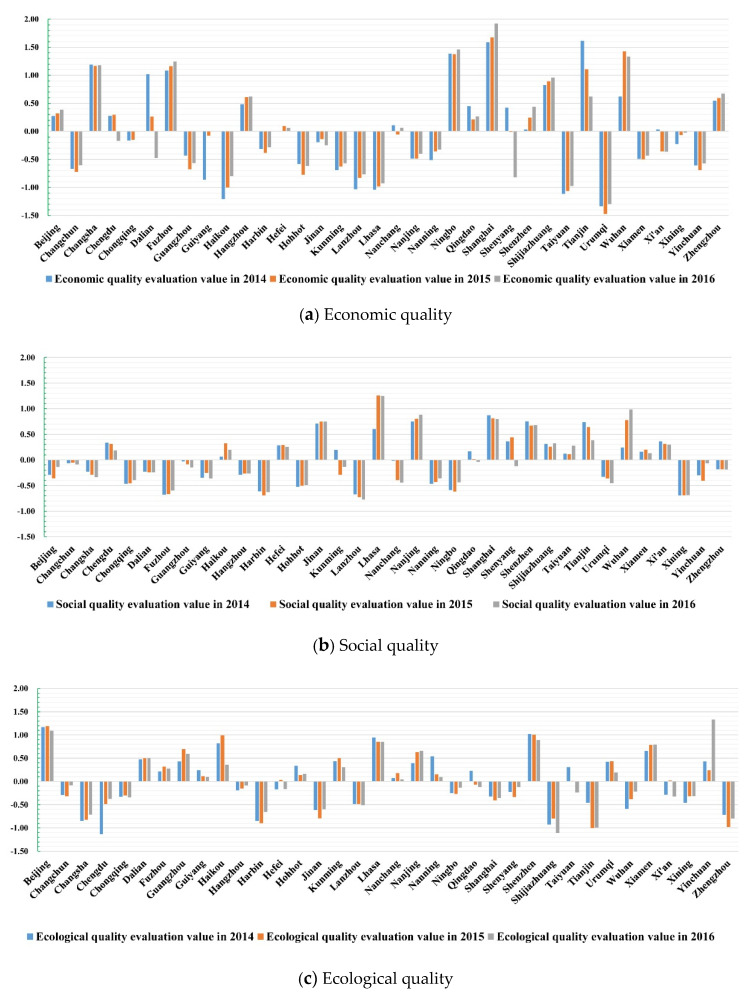

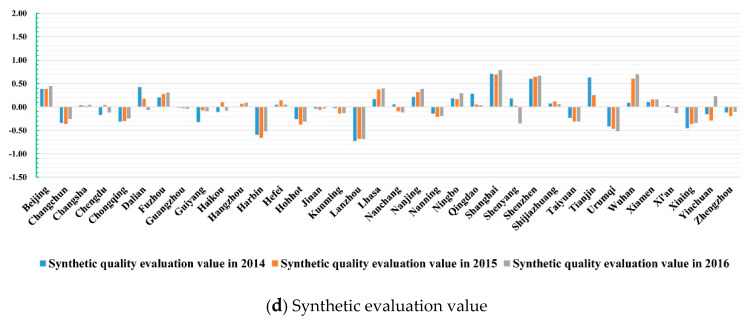
Bar chart of CLUQ evaluation in 2014–2016.

The synthetic evaluation model is used to calculate the CLUQ in 2014, 2015, and 2016; synthetic evaluation value of the three years (Figure 3) and its mean (Table 2) are obtained. According to the bar chart of CLUQ evaluation in 2014, 2015, and 2016, we can see the following: (1) From 2014 to 2016, the synthetic evaluation values of CLUQ in 13 cities, Dalian, Guangzhou, Hohhot, Kunming, Nanchang, Nanning, Qingdao, Shenyang, Shijiazhuang, Taiyuan, Tianjin, Urumqi, and Xi’an, decreased year by year, whereas that of other cities showed an increase trend even though it declined slightly in the middle of this period. (2) In terms of economic quality, half of the cities’ economic quality evaluation values were increasing. That value in these 8 cities, Harbin, Hefei, Xiamen, Shenzhen, Urumqi, Wuhan, Yinchuan, and Changchun, was rising generally with a slight drop in the middle. However, the remaining 10 cities, Chengdu, Dalian, Guangzhou, Hohhot, Nanchang, Qingdao, Shenyang, Tianjin, Xi’an, and Changsha, showed a decrease trend in land use quality. (3) In terms of social quality, only eight cities, namely, Hohhot, Jinan, Lhasa, Nanjing, Nanning, Wuhan, Xining, and Chongqing, were in a state of increasing social quality from 2014 to 2016. The social quality of eight cities, Beijing, Fuzhou, Haikou, Hangzhou, Ningbo, Shijiazhuang, Taiyuan, and Yinchuan, was in an overall growth state, with a slight decline in the middle; However, the social quality of the remaining 20 cities was in a declining trend. (4) In terms of ecological quality, only 7 cities, Dalian, Hangzhou, Nanjing, Xiamen, Wuhan, Xining, and Changsha, have increased their ecological quality year by year; the ecological quality of 9 cities, Fuzhou, Guangzhou, Harbin, Hefei, Jinan, Nanning, Ningbo, Yinchuan, and Changchun, has shown an overall upward trend, with a slight decrease in the middle; and the ecological quality of the remaining 20 cities is in a downward trend.

The development trend of CLUQ can reflect the local government’s investment and improvement in economic, social, and ecological quality. The synthetic quality of urban construction land use in most of the research objects is on the rise. It means the land-use policy of the local government can effectively promote the intensive use of construction land. The economic quality of urban construction land use in most of the research objects is on the rise. It is because the local government has carried out the main financial input and policy support in improving the economic quality of construction land due to the economic output being the main assessment index of the local government. However, in terms of social and ecological quality, more than half of the cities are in a decreasing state, which indicates that the local government has not invested enough in the ecological and social construction of construction land.

Through horizontal comparison of the economic quality, social quality, and ecological quality of construction land use in the synthetic quality (Table 3), it is demonstrated that the economic quality of construction land use in 14 cities, Beijing, Fuzhou, Harbin, Hangzhou, Ningbo, Qingdao, Shanghai, Shijiazhuang, Tianjin, Wuhan, Xining, Changsha, Zhengzhou, and Chongqing, is higher than the social and ecological quality of construction land use, and they are in the dominant position. In Chengdu, Hefei, Jinan, Nanjing, Lhasa, Shenyang, Taiyuan, Xi’an, and Changchun, the social quality is higher than the economic and ecological quality; the ecological quality of construction land use in the remaining 13 cities is higher than the economic and social quality, which are in the dominant position.

**Figure 4:**
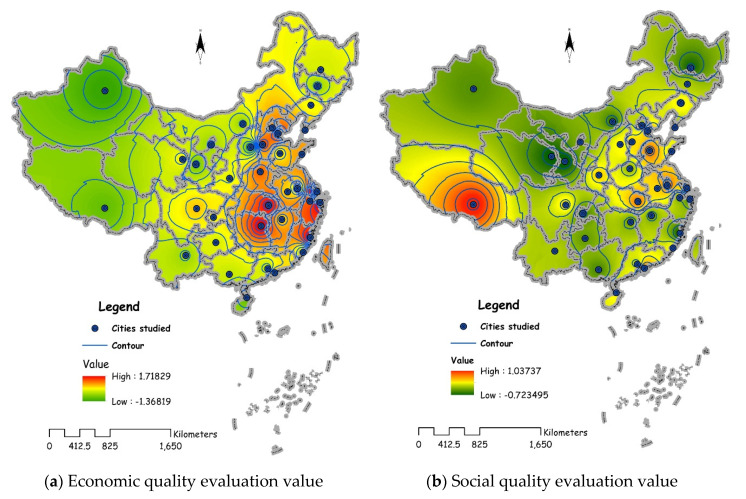

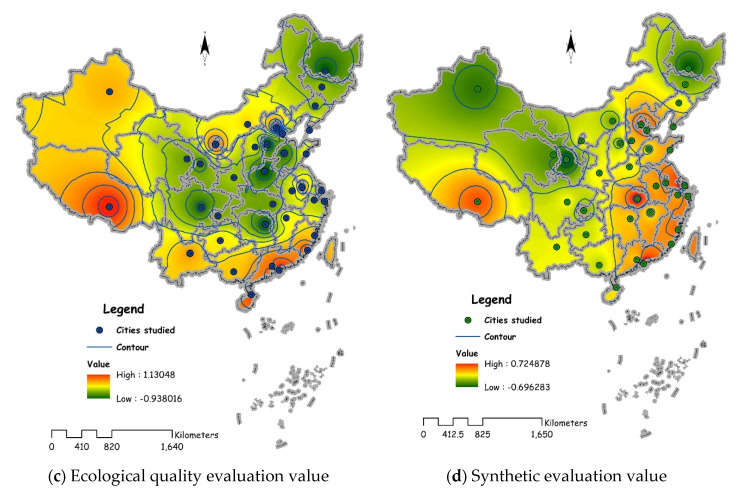
Contour map of urban CLUQ across China.

In order to map the spatial characteristics of CLUQ, the inverse distance weight method (IDW) is used to interpolate the data based on the ArcGIS 10.2 software platform (Figure 4). In terms of synthetic quality, the synthetic quality of urban construction land use in eastern and southwest China is higher; in terms of spatial differences, the economic quality of eastern and central cities is the dominant factor in the use of construction land, and the social quality of eastern, northern, and southwestern cities is the dominant factor in the use of construction land. In the west, southwest and north of China, the ecological quality is the dominant factor of urban construction land use.

**Figure 5:**
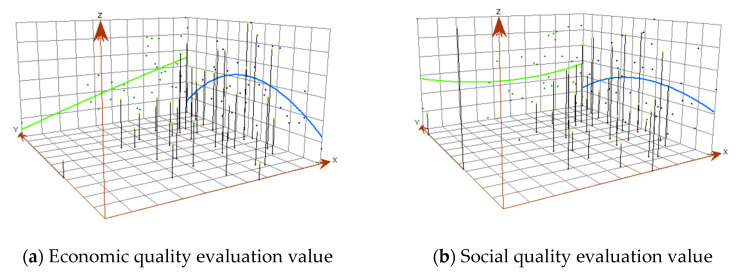

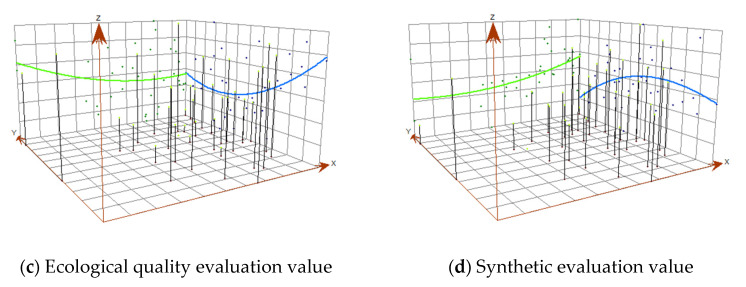
Trend analysis of evaluation value of urban CLUQ.

To further analyze the spatial trend characteristics of construction land use quality, the ArcGIS 10.2 “trend analysis” tool is used to draw the trend analysis chart of each subsystem and synthetic evaluation value (Figure 5). It can be seen from Figure 5a that in the longitude, the economic quality of construction land use gradually increases from west to east. While in the latitude, it firstly increases from north to south and then decreases. It forms an inverted U curve. It can be seen from Figure 5b that in the longitude, the social quality of the construction land use first decreases from west to east and then increases, and the overall state is relatively stable, while in the latitude, it presents the inverted U state. In Figure 5c, the ecological quality of construction land use decreases from west to east in longitude and decreases first and then increases in latitude. It shows the synthetic quality of construction land use is increasing from west to east in longitudinal direction and presents an inverted U-shaped state in latitudinal direction (Figure 5d).

### 4.2. Coupling Degree of Urban Construction Land in China

The coupling evaluation model is used to calculate the coupling degree of economic quality, social quality, and ecological quality of construction land use, and the results are shown in Table 4.

**Figure 6:**
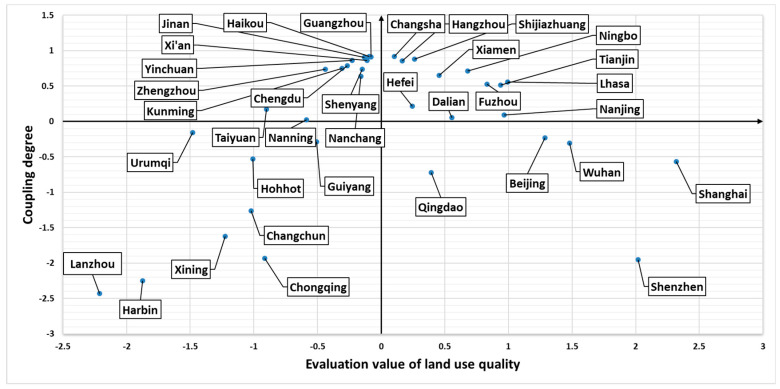
The coordinate system of CLUQ-CD.

To intuitively express the relationship between the coupling degree and quality of urban construction land use, we use the method of quadrant analysis to set the coupling degree and quality synthetic evaluation value as the *X*-axis and *Y*-axis respectively and mark them on the cartesian coordinate system. To make the synthetic evaluation value of coupling degree and quality clearly displayed in the cartesian coordinate system, *Z*-score standardization is firstly carried out for the results of the coupling degree and quality evaluation value in the study, and the specific calculation process is shown in Formula (1). The results of the quadrant analysis are shown in Figure 6.

Through the analysis of Figure 6, objects are divided into four categories: “high quality-high coupling”, “high quality-low coupling”, “low quality-high coupling”, and “low quality-low coupling”. Among them, 11 cities, Changsha, Hangzhou, Xiamen, Shijiazhuang, Ningbo, Hefei, Dalian, Nanjing, Lhasa, Tianjin, and Fuzhou, belong to the “high quality-high coupling” region, which needs to continue to maintain this good development trend; 12 cities, including Guangzhou, Haikou, Jinan, Yinchuan, Chengdu, Kunming, Zhengzhou, Shenyang, Nanning, Nanchang, Xi’an, and Taiyuan, belong to the “high coupling-low quality” area; although the coupling degree of the above areas is high, the low-level coordination still needs to improve the quality of construction land use; the five cities of Qingdao, Beijing, Wuhan, Shanghai, and Shenzhen are “high quality-low coupling” cities. Although the quality of construction land use in the above areas is high, there is no internal coordination, so there is still a large space for improvement with the improvement of internal coupling; the eight cities of Urumqi, Hohhot, Guiyang, Changchun, Xining, Chongqing, Harbin, and Lanzhou belong to the “low quality–low coupling” area, so the above areas need to improve both the coupling degree and the quality of construction land use.

### 4.3. Obstacle Diagnosis

To judge the dominant obstacle factors for improving the quality of construction land use, the obstacle diagnosis model is used to analyze the CLUQ in major cities of China, and the results are shown in Table 5.

Combined with the above evaluation results of CLUQ and coupling degree calculation, the study mainly diagnoses the obstacle degree of cities with a low coupling degree, and the results are shown in Table 3. The main obstacle to the improvement of the quality of construction land use in the five cities of Hohhot, Lanzhou, Urumqi, and Changchun is economic factors; the dominant obstacle in the five cities of Beijing, Guiyang, Shanghai, Xining, and Chongqing is social factors; the dominant obstacle in Harbin, Qingdao, and Wuhan is ecological factors.

## 5. Conclusions and Policy Implications

This study focuses on an analysis of the quality evaluation, spatial analysis, and obstacle diagnosis of CLUQ in 36 major cities in China based on ECO model. Through the analysis of the results, the following conclusions were drawn: First, from 2014 to 2016, the synthetic CLUQ in most cities showed an increasing state, among which, the economic quality of most cities showed an increase, and the social and ecological quality showed a decrease. Second, in terms of spatial characteristics, the synthetic CLUQ in eastern China and southwest China is relatively high. In terms of spatial trend, the synthetic CLUQ in the longitudinal direction was increasing from west to east, showing an inverted U-shaped state in the latitudinal direction. Third, in cities with low coupling degree such as Hohhot, Lanzhou, Urumqi, and Changchun, the main obstacle factor for improving the quality of construction land use is economic development; for Beijing, Guiyang, Shanghai, Xining, and Chongqing, it is social development, and for Harbin, Qingdao, and Wuhan, it is ecological development.

The improvement of CLUQ lies in the coupling and coordinated development of economic, social, and ecological quality. For cities with low coupling degree, we should analyze the location of quadrant and distinguish different types. Moreover, we should combine the diagnosis results of obstacle degree and implement effective practice for specific obstacle factors. For the “high quality–high coupling” type cities, the urban CLUQ is relatively high, and the internal coordination is good. We should maintain the coordination state to achieve the continuous improvement of the quality of construction land use. For “high quality–low coupling” cities, the CLUQ is higher, but this is the high quality with the low coupling. They have a large gap to improve the quality. We should eliminate the coupling obstacle factors to promote the CLUQ according to the result of obstacle degree diagnosis. For “low quality–low coupling”, both CLUQ and its internal coupling degree need to be improved. For “low quality–high coupling”, although the coupling degree is high, the coordination level is low. Therefore, the economic, social, and ecological quality of construction land use needs to be improved synergistically so that we can improve the synthetic construction land use quality.

The highlights of this study are: (1) ECO Model was constructed to evaluate construction land use quality. (2) Framework of construction land use quality was optimized from an economic-social-ecological coupling perspective. (3) Distribution and trend analysis mapping were drawn with the help of construction land use quality 3-D visualization. (4) A total of 36 main cities in China were identified into four types on construction land use quality spatial feature. (5) Joint management strategies were formulated for each type based on obstacle factors. It should be noted that there are some limitations: The study constructs an innovative framework and integration model, but the model is not perfect because of the different economic and social conditions of the world’s cities, as the development level of cities varies greatly, so it may be necessary to use this analytical framework with some parameter adjustments. At present, the research only takes provincial capital and above level cities in China as research objects to apply the developed model. Some small and medium-sized cities will be further explored and analyzed, and an improved multi-scale integration model will be of significance to establish.

## Figures and Tables

**Table 1:** Summary statistics of the variables.

Index	Units	Mean	Std. Dev	Min	Max
GDP per area	10,000 RMB/km^2^	14,141,720	5,233,156	3,667,159	28,096,850
Fixed asset investment per area	10,000 RMB/km^2^	102,819	42,522	30,533	204,671
Financial income per area	10,000 RMB/km^2^	16,902	9696	6587	64,125
Coverage ratio of community service institutions	Pcs/km^2^	0.03	0.02	0.0023	0.10
Per capita urban road area	m^2^/people	12.95	3.42	5.44	22.17
Per capita built-up area	km^2^/10,000 people	0.66	0.40	0.25	2.75
Per capita ecological land area	m^2^/people	26.17	11.42	11.54	72
Green coverage ratio of built-up area	%	41.09	5.49	25.50	61.58
PM2.5 annual average concentration	Microgram/m^3^	55.75	19.37	21	124

**Table 2:** Framework of the synthetic construction land use quality evaluation.

Criterion Level	Index Level	Index	Unit	Attribute	Literature Sources
	Land output	GDP per area	10,000 RMB/km^2^	Positive indicators	[19,47]
**Economic**	Land input	Fixed asset investment per area	10,000 RMB/km^2^	Positive indicators	[48,49]
	Revenue	Financial income per area	10,000 RMB/km^2^	Positive indicators	[50]
	Health service	Coverage ratio of community service institutions	Pcs/km^2^	Positive indicators	[12]
**Social**	Traffic	Per capita urban road area	m^2^/people	Positive indicators	[51,52]
	Living space	Per capita built-up area	km^2^/10,000 people	Positive indicators	[53,54]
	Ecological space	Per capita ecological land area	m^2^/people	Positive indicators	[55]
**Ecological**	Ecological structure	Green coverage ratio of built-up area	%	Positive indicators	[56]
	Environmental Health	PM2.5 annual average concentration	Microgram/m^3^	Negative index	[57,58]

**Table 3:** Average CLUQ evaluation value of 2014–2016.

City	Economic Quality	Social Quality	Ecological Quality	Synthetic Quality	City	Economic Quality	Social Quality	Ecological Quality	Synthetic Quality
**Beijing**	0.3274	−0.2625	1.1516	0.4055	**Ningbo**	1.4079	−0.5479	−0.2162	0.2146
**Chengdu**	0.1332	0.2791	−0.6643	−0.0840	**Qingdao**	0.3098	0.0479	0.0136	0.1238
**Dalian**	0.2700	−0.2379	0.4945	0.1755	**Xiamen**	−0.4758	0.1644	0.7424	0.1437
**Fuzhou**	1.1627	−0.6459	0.2698	0.2622	**Shanghai**	1.7284	0.8261	−0.3606	0.7313
**Guangzhou**	−0.5587	−0.0873	0.5723	−0.0246	**Shenzhen**	0.2380	0.7013	0.9706	0.6366
**Guiyang**	−0.3108	−0.3234	0.1535	−0.1602	**Shenyang**	−0.1385	0.2270	−0.2281	−0.0465
**Harbin**	−0.3288	−0.6444	−0.8000	−0.5911	**Shijiazhuang**	0.8931	0.2990	−0.9433	0.0829
**Haikou**	−1.0037	0.1940	0.7215	−0.0294	**Taiyuan**	−1.0504	0.1687	0.0301	−0.2839
**Hangzhou**	0.5731	−0.2739	−0.1421	0.0524	**Tianjin**	1.1168	0.5875	−0.8181	0.2954
**Hefei**	0.0545	0.2762	−0.0995	0.0771	**Urumqi**	−1.3682	−0.3827	0.3500	−0.4670
**Hohhot**	−0.6579	−0.5066	0.2118	−0.3176	**Wuhan**	1.1283	0.6663	−0.3954	0.4664
**Jinan**	−0.1932	0.7345	−0.6668	−0.0418	**Xi’an**	−0.2297	0.3232	−0.1971	−0.0345
**Kunming**	−0.6298	−0.0770	0.4152	−0.0972	**Xining**	−0.1051	−0.6902	−0.3640	−0.3864
**Lhasa**	−0.9838	1.0375	0.8846	0.3128	**Yinchuan**	−0.6252	−0.2579	0.6670	−0.0720
**Lanzhou**	−0.8766	−0.7241	−0.4905	−0.6971	**Changchun**	−0.6659	−0.0698	−0.2303	−0.3220
**Nanchang**	0.0378	−0.2847	0.0969	−0.0500	**Changsha**	1.1769	−0.2844	−0.7944	0.0327
**Nanjing**	−0.4578	0.8107	0.5607	0.3045	**Zhengzhou**	0.6033	−0.1841	−0.8340	−0.1382
**Nanning**	−0.3981	−0.4191	0.2618	−0.1851	**Chongqing**	−0.1029	−0.4397	−0.3235	−0.2887

**Table 4:** Coupling degree (CD) of urban CLUQ system.

City	CD	City	CD	City	CD	City	CD
Beijing	0.672	Hefei	0.800	Ningbo	0.941	Urumqi	0.694
Chengdu	0.961	Hohhot	0.588	Qingdao	0.533	Wuhan	0.652
Dalian	0.753	Jinan	0.995	Xiamen	0.923	Xi’an	0.982
Fuzhou	0.888	Kunming	0.951	Shanghai	0.578	Xining	0.277
Guangzhou	0.997	Lhasa	0.896	Shenzhen	0.184	Yinchuan	0.983
Guiyang	0.657	Lanzhou	0.049	Shenyang	0.947	Changchun	0.380
Harbin	0.099	Nanchang	0.918	Shijiazhuang	0.988	Changsha	0.998
Haikou	0.998	Nanjing	0.764	Taiyuan	0.787	Zhengzhou	0.948
Hangzhou	0.981	Nanning	0.745	Tianjin	0.884	Chongqing	0.190

**Table 5:** Obstacle degree of economic, social, and ecological subsystem.

City	Economic Obstacle	Social Obstacle	Ecological Obstacle	City	Economic Obstacle	Social Obstacle	Ecological Obstacle
Beijing	0.3771	0.7079	−0.0850	Ningbo	−0.1731	0.6569	0.5162
Chengdu	0.2665	0.2217	0.5118	Qingdao	0.2626	0.3622	0.3752
Dalian	0.2951	0.5005	0.2044	Xiamen	0.5745	0.3253	0.1003
Fuzhou	−0.0735	0.7436	0.3299	Shanghai	−0.9037	0.2158	1.6879
Guangzhou	0.5071	0.3537	0.1392	Shenzhen	0.6991	0.2740	0.0269
Guiyang	0.3766	0.3802	0.2432	Shenyang	0.3626	0.2462	0.3912
Harbin	0.2784	0.3445	0.3771	Shijiazhuang	0.0389	0.2548	0.7063
Haikou	0.6488	0.2610	0.0902	Taiyuan	0.5323	0.2158	0.2518
Hangzhou	0.1502	0.4481	0.4017	Tianjin	−0.0552	0.1952	0.8601
Hefei	0.3415	0.2614	0.3971	Urumqi	0.5381	0.3142	0.1477
Hohhot	0.4194	0.3812	0.1994	Wuhan	−0.0801	0.2084	0.8717
Jinan	0.3818	0.0849	0.5333	Xi’an	0.3962	0.2181	0.3857
Kunming	0.4951	0.3272	0.1777	Xining	0.2657	0.4064	0.3279
Lhasa	0.9622	−0.0182	0.0560	Yinchuan	0.5053	0.3911	0.1035
Lanzhou	0.3686	0.3386	0.2928	Changchun	0.4201	0.2697	0.3102
Nanchang	0.3055	0.4078	0.2867	Changsha	−0.0610	0.4426	0.6184
Nanjing	0.6987	0.0907	0.2105	Zhengzhou	0.1162	0.3468	0.5371
Nanning	0.3932	0.3991	0.2076	Chongqing	0.2853	0.3724	0.3423

## Supplementary Materials

The following are available online at https://www.mdpi.com/1660-4601/17/10/3663/s1, Table S1: Average CLUQ evaluation value of 2014–2016 using entropy weight method, Table S2: Average CLUQ evaluation value of 2014–2016 using equal weight method.
