# Supplementary material for: An Exploration of a Synthetic Construction Land Use Quality Evaluation Based on Economic-Social-Ecological Coupling Perspective: A Case Study in Major Chinese Cities
Source: Int J Environ Res Public Health. 2020 May 22;17(10):3663. doi: 10.3390/ijerph17103663 (PMC7277519; doi:10.3390/ijerph17103663)
Supplement: Supplementary file 1 [file ijerph-17-03663-s001.pdf]

An exploration of a synthetic construction land use quality evaluation based on economic-social-ecological coupling perspective: a case study in major Chinese cities

Xufeng Cui[1], Sheng Yang[1], Guanghong Zhang[1*], Bin Liang[2], Fei Li[3]**

School of Business Administration, Zhongnan University of Economics and Law, Wuhan 430073, China; cxf@zuel.edu.cn (X.C.); 201811080234@stu.zuel.edu.cn (S.Y.)

[2] Development and Rural Innovation, Wageningen University and Research, Wageningen 6709PH, The Netherlands; bin.liang@wur.nl

[3] School of Information and Safety Engineering, Zhongnan University of Economics and Law, Wuhan 430073, China

Supplementary information

In the process of research, the equal weight method and entropy weight method were involved in the model calculation as the index weight calculation method respectively. The results of entropy weight method show that the fitting degree of realistic land use was relatively low, because entropy weight method use "degree of dispersion" of the data to calculate the importance of data, the greater the degree of dispersion of the index, the greater the weight of the index, the importance of indicators in reality is not taken into account, while construction land use quality is closely related to the realistic land use. The output of the model driven by equal weight method fits well with reality. At the same time, we also verified this result by consulting many experts, so we choose the equal weight method as the calculation method of index weights. The comparison results of the two methods are as follows.

Here we report the results of entropy weight method and equal weight method. The weights of each index obtained by entropy weight method are: 0.103, 0.149, 0.748, 0.347, 0.171, 0.482, 0.699, 0.179, and 0.122. Then, the average evaluation value of CLUQ in 2014-2016 using entropy weight method is shown in Table S1.

Table S1. Average CLUQ evaluation value of 2014-2016 using entropy weight method.

City	Economic quality	Social quality	Ecological quality	Synthetic quality	City	Economic quality	Social quality	Ecological quality	Synthetic quality
**Beijing**	1.1063	-0.0348	1.2911	0.7876	**Ningbo**	1.4038	-0.5260	-0.6183	0.0865
**Chengdu**	0.0405	0.2696	-0.7195	-0.1365	**Qingdao**	0.2011	-0.0438	-0.0727	0.0282
**Dalian**	0.0926	-0.1721	0.4051	0.1085	**Xiamen**	-0.0628	0.2531	0.7087	0.2997
**Fuzhou**	0.7483	-0.6854	-0.4337	-0.1236	**Shanghai**	3.0224	0.8782	-0.6771	1.0745
**Guangzhou**	-0.5984	0.1601	0.7069	0.0896	**Shenzhen**	0.8007	0.7734	0.8871	0.8204
**Guiyang**	-0.3194	-0.2146	-0.0770	-0.2037	**Shenyang**	-0.1949	0.0119	-0.2132	-0.1321
**Harbin**	-0.5741	-0.7013	-0.8889	-0.7214	**Shijiazhuang**	0.1654	-0.1564	-1.0083	-0.3331
**Haikou**	-0.9851	0.3227	0.3735	-0.0963	**Taiyuan**	-0.9667	0.2240	0.3416	-0.1337
**Hangzhou**	0.6243	-0.3025	-0.2127	0.0364	**Tianjin**	1.2986	0.4258	-0.6641	0.3534

**Hefei**	-0.1951	0.1531	-0.1106	-0.0509	**Urumqi**	-1.0576	0.0624	1.2115	0.0721
**Hohhot**	-0.7228	-0.3214	0.3128	-0.2438	**Wuhan**	0.9422	0.2746	-0.4807	0.2454
**Jinan**	-0.2212	0.3372	-0.4222	-0.1021	**Xi'an**	-0.3239	0.1609	-0.1733	-0.1121
**Kunming**	-0.5370	-0.1625	0.1237	-0.1919	**Xining**	-0.4941	-0.6922	-0.6818	-0.6227
**Lhasa**	-0.6911	1.8800	2.3906	1.1932	**Yinchuan**	-0.6454	-0.2395	0.7747	-0.0368
**Lanzhou**	-0.9085	-0.5547	-0.4542	-0.6391	**Changchun**	-0.8143	-0.1770	-0.1382	-0.3765
**Nanchang**	-0.2229	-0.2608	-0.1208	-0.2015	**Changsha**	0.6122	-0.3433	-0.8404	-0.1905
**Nanjing**	-0.4044	0.6352	0.9654	0.3987	**Zhengzhou**	0.5210	-0.1448	-0.7036	-0.1091
**Nanning**	-0.5714	-0.5843	-0.2294	-0.4617	**Chongqing**	-0.0685	-0.5048	-0.5521	-0.3752

And the average evaluation value of CLUQ in 2014-2016 using equal weight method is shown in Table S2.

Table S2 Average CLUQ evaluation value of 2014-2016 using equal weight method

City	Economic quality	Social quality	Ecological quality	Synthetic quality	City	Economic quality	Social quality	Ecological quality	Synthetic quality
**Beijing**	0.3274	-0.2625	1.1516	0.4055	**Ningbo**	1.4079	-0.5479	-0.2162	0.2146
**Chengdu**	0.1332	0.2791	-0.6643	-0.0840	**Qingdao**	0.3098	0.0479	0.0136	0.1238
**Dalian**	0.2700	-0.2379	0.4945	0.1755	**Xiamen**	-0.4758	0.1644	0.7424	0.1437
**Fuzhou**	1.1627	-0.6459	0.2698	0.2622	**Shanghai**	1.7284	0.8261	-0.3606	0.7313
**Guangzhou**	-0.5587	-0.0873	0.5723	-0.0246	**Shenzhen**	0.2380	0.7013	0.9706	0.6366
**Guiyang**	-0.3108	-0.3234	0.1535	-0.1602	**Shenyang**	-0.1385	0.2270	-0.2281	-0.0465
**Harbin**	-0.3288	-0.6444	-0.8000	-0.5911	**Shijiazhuang**	0.8931	0.2990	-0.9433	0.0829
**Haikou**	-1.0037	0.1940	0.7215	-0.0294	**Taiyuan**	-1.0504	0.1687	0.0301	-0.2839
**Hangzhou**	0.5731	-0.2739	-0.1421	0.0524	**Tianjin**	1.1168	0.5875	-0.8181	0.2954
**Hefei**	0.0545	0.2762	-0.0995	0.0771	**Urumqi**	-1.3682	-0.3827	0.3500	-0.4670
**Hohhot**	-0.6579	-0.5066	0.2118	-0.3176	**Wuhan**	1.1283	0.6663	-0.3954	0.4664
**Jinan**	-0.1932	0.7345	-0.6668	-0.0418	**Xi'an**	-0.2297	0.3232	-0.1971	-0.0345
**Kunming**	-0.6298	-0.0770	0.4152	-0.0972	**Xining**	-0.1051	-0.6902	-0.3640	-0.3864
**Lhasa**	-0.9838	1.0375	0.8846	0.3128	**Yinchuan**	-0.6252	-0.2579	0.6670	-0.0720
**Lanzhou**	-0.8766	-0.7241	-0.4905	-0.6971	**Changchun**	-0.6659	-0.0698	-0.2303	-0.3220
**Nanchang**	0.0378	-0.2847	0.0969	-0.0500	**Changsha**	1.1769	-0.2844	-0.7944	0.0327
**Nanjing**	-0.4578	0.8107	0.5607	0.3045	**Zhengzhou**	0.6033	-0.1841	-0.8340	-0.1382
**Nanning**	-0.3981	-0.4191	0.2618	-0.1851	**Chongqing**	-0.1029	-0.4397	-0.3235	-0.2887

It can be seen from the results that in terms of the synthetic quality evaluation value. In the result of entropy weight method, the synthetic quality evaluation value of Lhasa in China is the first, and the synthetic evaluation value of Yinchuan, Kunming, Hohhot and other underdeveloped areas is higher than that of developed cities in China, which is not consistent

with the reality. In addition, according to the weight value obtained by entropy weight method, it can be seen that the third and seventh indicators are too large compared with other indicators, which are the financial income per area and the per capita ecological land area, but They cannot be more than twice as important as other indicators, so the results of entropy weight method are inconsistent with the actual situation. The results obtained by using equal weight method are consistent with the actual situation. Therefore, compared with the entropy weight method, this study finally employed the equal weight method.
